# Supplementary material for: First Evidence of “Earth Wax” Inside the Casting Molds from the Roman Era
Source: Molecules. 2021 Jul 13;26(14):4259. doi: 10.3390/molecules26144259 (PMC8306946; doi:10.3390/molecules26144259)
Supplement: Supplementary file 1 [file molecules-26-04259-s001.zip › molecules-1250250-supplementary.pdf]

First Evidence of Earth Wax Inside the Casting Molds from the Roman Era

Klára Jagošová, Jan Jílek, Pavel Fojtík, Ivan Čižmář, Miroslav Popelka, Ondřej Kurka and Lukáš Kučera

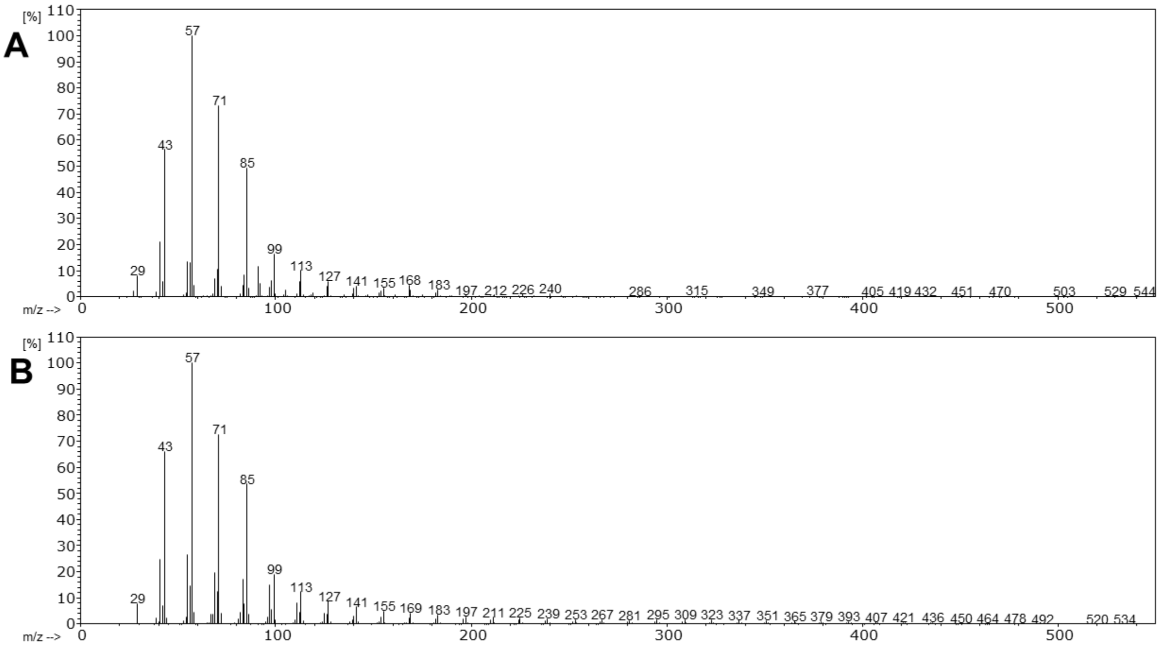

Figure S1: Combined mass spectra of all detected peaks in pyrogram (range 8.0–19.0 min) of the solid sample from mold #2 (**A**) and the database reference mass spectrum of ozokerite wax C1-C40 (**B**)

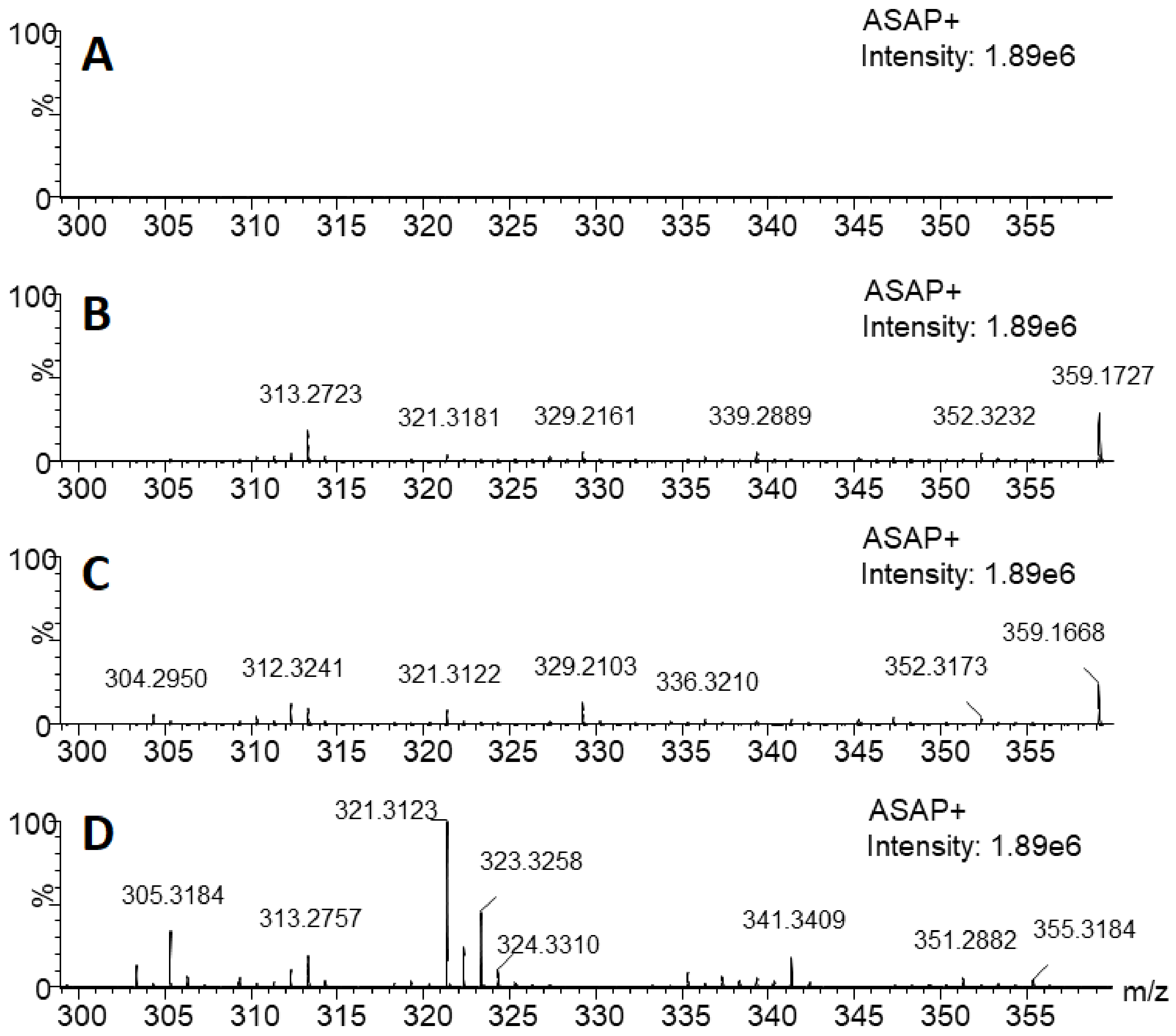

Figure S2: ASAP-IMS-HRMS spectrum in MS mode of blank (**A**), sample from mold #1 (**B**), mold #2 (**C**) and ceresin standard (**D**) in mass range 300-360 Da.
